# Comment on: Oxytocin: a neglected hormone in pituitary disease – From function to the diagnosis of a deficiency, resulting clinical relevance, and potential treatment options in endocrinology

**DOI:** 10.20945/2359-4292-2026-0025

**Published:** 2026-03-19

**Authors:** Aleena Elia

**Affiliations:** 1 Liaquat University of Medical and Health Sciences (LUMHS), Jamshoro, Sindh, Pakistan

Dear Editor,

I read with great interest the recent article by Sevenja Leibnitz and cols., entitled “Oxytocin: a neglected hormone in pituitary disease – From function to the diagnosis of a deficiency, resulting clinical relevance, and potential treatment options in endocrinology” ^(1)^.

The authors provide an extensive and perceptive analysis of oxytocin, emphasizing its frequently overlooked functions beyond reproduction. The study highlights the potential significance of oxytocin deficiency in pituitary dysfunction by effectively integrating molecular, physiological, and clinical perspectives. However, I would like to highlight a few points to strengthen the research.

First, while the study relies extensively on animal research to understand the role of oxytocin, these models provide important foundational insights. Future human research could expand on this strong preclinical work to validate and translate these findings, thereby improving our understanding of oxytocin’s role in pituitary dysfunction ^(2)^.

Although the research provides a thorough overview of oxytocin physiology, future research could strengthen the topic by accounting for sex differences. Since oxytocin biology and receptor expression fluctuate between males and females, as well as across different life stages, integrating these factors may help elucidate discrepancies in clinical presentation and treatment response ^(3)^.

Moreover, the study emphasizes the possible involvement of oxytocin insufficiency in pituitary disorders; yet, subsequent research could enhance these conclusions by accounting for prevalent comorbidities in hypopituitary patients. A more comprehensive approach could help illuminate the independent role of oxytocin in clinical symptoms, as many people have numerous hormone deficits and metabolic problems ^(4)^.

Lastly, the authors meticulously inspect analyses the therapeutic potential of intranasal oxytocin. However, future research could further strengthen this domain by concentrating on long-term safety, appropriate dose, and probable interactions with other hormone replacement therapies. Well-designed clinical trials will be desired to show that oxytocin-based treatments are both effective and safe before widespread clinical use ^(5)^.

In conclusion, the authors’ perceptive analysis emphasizes the significant but frequently disregarded function that oxytocin plays in pituitary disorders. Future research will advance our knowledge of oxytocin and the potential clinical uses by addressing areas concerning human validation, sex variations, co-morbidities, and long-term therapeutic safety.

## Data Availability

the datasets used and/or analyzed during the current study available from the corresponding author on rea-sonable request.
